# Analysis of Stability, Rheological and Structural Properties of Oleogels Obtained from Peanut Oil Structured with Yellow Beeswax

**DOI:** 10.3390/gels8070448

**Published:** 2022-07-18

**Authors:** Anna Zbikowska, Sylwia Onacik-Gür, Małgorzata Kowalska, Michał Sowiński, Iwona Szymańska, Katarzyna Żbikowska, Katarzyna Marciniak-Łukasiak, Wojciech Werpachowski

**Affiliations:** 1 Institute of Food Sciences, Faculty of Food Assessment and Technology, Warsaw University of Life Sciences (WULS-SGGW), Nowoursynowska st. 159c, 02-776 Warsaw, Poland; anna_zbikowska@sggw.edu.pl (A.Z.); michalkarlowski1998@gmail.com (M.S.); iwona_szymanska@sggw.edu.pl (I.S.); katarzyna_marciniak_lukasiak@sggw.edu.pl (K.M.-Ł.); 2 Department of Meat and Fat Technology, Prof. Waclaw Dąbrowski Institute of Agricultural and Food Biotechnology-State Research Institute, 36 Rakowiecka st., 02-532 Warsaw, Poland; sywia.onacik-gur@ibprs.pl; 3 Faculty of Chemical Engineering and Commodity Science, Kazimierz Pulaski University of Technology and Humanities, Chrobrego st. 27, 26-600 Radom, Poland; 4 Faculty of Medicine, Medical University of Warsaw, Zwirki i Wigury st. 61, 02-091 Warsaw, Poland; kasiazbikus@wp.pl; 5 Institute of Organization of Production Systems, Warsaw University of Technology, 85 Narbutta st, 02-524 Warsaw, Poland; wojciech.werpachowski@pw.edu.pl

**Keywords:** oleogelation, MS-DWS method, mechanical property, centrifugal stability analysis

## Abstract

The aim of this study was to evaluate the macro- and microscopic properties of oleogels with yellow beeswax using different methods, especially modern optical techniques. Microrheological properties, physical stability and morphology of oleogel crystals obtained by structuring of peanut oil with yellow beeswax was analyzed. It was observed that oleogels, even with the smallest concentration of beeswax (2%), were resistant to centrifugal force. Increase in yellow beeswax concentration (from 2, 4, 6 to 8 %) resulted in significant differences in the characteristics of oleogels: increased elasticity (EI), macroscopic viscosity (MVI) and the firmness values of oleogels. It was concluded that non-invasive optical techniques (multi-speckle diffusing wave spectroscopy—Rheolaser Master) are useful in obtaining a quick evaluation of physical properties of oleogels at the microstructural level, and the received information allows for quality assessment.

## 1. Introduction

Oleogels are obtained from oil with a small addition of oil structurants. They are produced by a physical method and combine the functional properties of solid fats with a nutritious profile of fatty acids (FAs), typical for liquid oils. The most commonly used substances for oleogelation are monomers such as plant and animal waxes, monoglycerides, fatty acids, fatty alcohols, sterols, lecithin and the polymers ethyl cellulose and HPMC [1]. Beeswax has very good oil-structuring properties. It is a natural wax and food-grade additive. Beeswax has a complex chemical composition. Its main compounds are hydrocarbons, wax esters, free fatty acids and long-chain alcohols [2].

Peanut oil is characterized by a high content of monounsaturated FA (approx. 72%), and polyunsaturated FA of approx. 18.5%. Oleic acid, which is the main fatty acid in peanut oil (approx. 71%), is valued for its positive effect on lowering the risk of cardiovascular diseases by lowering LDL cholesterol in blood [3]. Oils rich in oleic acid are resistant to oxidation, which is good for their storage properties [4]. Among polyunsaturated FAs, linoleic acid has the highest content (approx. 18.2%). This fatty acid belongs to the group of essential fatty acids, which plays many important biological functions in the human body. Polyunsaturated fatty acids help in preventing cardiovascular disease and coronary heart disease as well as hypertension, cancer, inflammatory diseases, autoimmune diseases, and diabetes type two [3].

Scientists see great potential in oleogels and various possibilities for their use. They are a promising alternative to solid food fats, which are undesirable for health reasons, used in food production [1,5]. They can be a carrier of biologically active substances (e.g., antioxidants) [6,7] and medicinal substances (capsaicin—used for nephropathic pain) [8]. They can be a gel base for active substances with an analgesic effect (rheumatic diseases) [9] or used in the treatment of wounds, as well as an alternative to traditional hydrophobic bases such as petroleum jelly (Vaselinum, Petrolatum) [10]. Oleogels can be designed to have a smooth and creamy consistency. It is very important in cases of patients who have a problem with swallowing tablets, especially children in the ages of 0–5 years old [11].

It has been shown that they are a good substrate for salicylic acid, cignolin or Peruvian balsam [12], an ointment base used in preparations for the treatment of diseases associated with epidermal keratosis disorders caused by incorrect lipid synthesis [13]. Oleogels are believed to provide better solubility of hydrophobic active substances. Ethanol is commonly used as a solvent. However, this substance is restricted in pediatric formulations. That is why oleogels could be an alternative for being a carrier of water-insoluble drugs [11]. Due to their lipophilic nature, they can also increase the penetration of some active compounds through the stratum corneum [13,14,15]. They are more resistant to microbial contamination compared to hydrogels and emulsions, and therefore do not require the addition of preservatives.

The usage of different techniques in oleogels analysis allows one to determine their structure and properties [16,17]. The results obtained by the instrumental measurement of texture parameters constitute a basis for inference about the potential use of oleogels, e.g., in the food industry or in pharmacy [17,18].

The optical microscopy technique is commonly used in analysis of structured lipids [18,19]. This technique allows the characterization of fats and lipid crystals, their distribution, size and shape. Analysis of microscopic images of crystals developed in oleogels allows for preliminary detection and identification of oleogelators [20]. Another optical technique which measures light transmission and scattering by the sample provides important information on microrheological properties of the tested samples [21]. Usually, the rheological test makes measurements on the macroscopic scale and damages samples. The MS-DWS (Multi-Speckle Diffusing Wave Spectroscopy) method is an alternative. The MS-DWS method tracks changes in particle motion under the influence of thermal energy in real time (through dynamic light scattering). The advantage of the passive optical microrheology technique is its low invasiveness, and the same sample can be again used for analysis [21,22,23].

In the MS-DWS method, microrheological parameters are calculated based on the mean square displacement of a particle at different frequencies as a function of time [24,25]. Usually, this technique is used for emulsion and hydrogel analysis [21,23,25]; however, application of this method to study oleogels is a novel way of characterizing these lipid systems. The aim of this study was to analyze the macro- and microscopic properties of oleogels with yellow beeswax at concentrations of 2, 4, 6 and 8% using known methods and modern optical techniques.

## 2. Results and Discussion

### 2.1. Analysis of Stability, Texture and Microrheological Properties

Oleogel with 2% of yellow beeswax was at the critical concentration. It had semi-solid consistency. It was reported that oleogel with beeswax at a critical concentration of 3% was formed in camellia, soybean and sunflower oils. The ability of the oleogelator to hold in its crystal network depends also on the fatty acid composition of oil. Beeswax oleogels form stronger gels in oils rich in long chain triglycerides [26]. Peanut oil is rich in long chain fatty acids, which possibly helps to form oleogels at lower concentrations of oleogelator.

All samples, irrespective of the amount of oleogelator, were very stable during the centrifuge test (Table 1) and did not differ statistically significantly (*p* ≤ 0.05). Therefore, it can be concluded that even the smallest addition of yellow beeswax (2%) allowed to obtain an oleogel with very good stability. Similarly, high stability of oleogels from grapeseed oil and beeswax (in concentrations of 5, 10 and 15%), exceeding 99%, was obtained by Yi et al. [27]. Other authors have confirmed the significant influence of the amount and type of gelling agent on the stability [18,28].

Textural and rheological properties of oleogels (e.g., creaminess, softness, plasticization, aeration, etc.) determine their further application [18,29,30]. Results of all samples were statistically significant (*p* ≤ 0.05). It was shown that the amount of yellow beeswax addition had a statistically significant effect on all texture parameters. Other scientists have also come to similar conclusions [31,32]. It was found that oleogels with 8% of wax content were characterized by a firmness of approx. 14 N and a work of shear of approx. 16 N mm. Values of these parameters for samples with 2% of yellow beeswax were more than 16 and 24 times lower (respectively). In addition, as the wax content increased, there is lower stickiness and spreadability of oleogel. Other studies arrived at similar conclusions [21,33]. The average values of firmness and stickiness indicate good lubricity of the product [33,34]. The lubricity is a very important parameter in the case of many food products [33,34], but also medications in the form of ointments, creams and suppositories.

EI and MVI values rose with the increase in yellow beeswax concentration, (Table 1), which proves enhancement of elastic properties of oleogels, which is typical for solids [21]. This phenomenon can be explained by the fact that, in samples with a higher concentration of the additive, the particles are more tightly packed. In oleogel, the oil has been closed in the 3D crystal network of the “gelificator”. At a higher concentration of the oleogelator, the network is denser. According to Pasqua et al. [35], the more tightly the particles are packed, the greater the elasticity, and thus the value of the elasticity index increases. Cristiano et al. [36] describe the dependence that the slower the movement of molecules, the higher the sample viscosity. In oleogels, the movement of the particles is slowed down by the three-dimensional network of the stabilizing substance, which is much denser at a higher concentration of beeswax additive. Similarly, Zhua et al. [37] showed that the increase in gelatin concentration in the double emulsion (w/o/w) resulted in an increase in the EI and MVI values. Oil-gelling substances significantly influence rheological properties of oleogels, which was confirmed by other authors using well known analyses [38,39]. Scientists showed a positive correlation between values of elastic modulus G’ and hardness and waxes concentration (candelilla, rice bran, yellow bees, carnauba). The increase in wax addition in oleogel results in greater aggregation of crystals and a denser network [38]. Passive microrheology enables the monitoring of rheological behavior and stability of highly concentrated systems based on structural changes on a microscopic scale. It was found that it can help explain the mechanisms responsible for such changes in gels and emulsions, which are usually heterogeneous [40,41]. Microrheological measurements (based on thermal movement of particles) and macroscopic measurements of mechanical properties (requiring external force, e.g., mechanical shear) complement each other in determining the detailed characteristics of gel systems [42].

### 2.2. Analysis of Microscopic Observations

**Figure 1:**
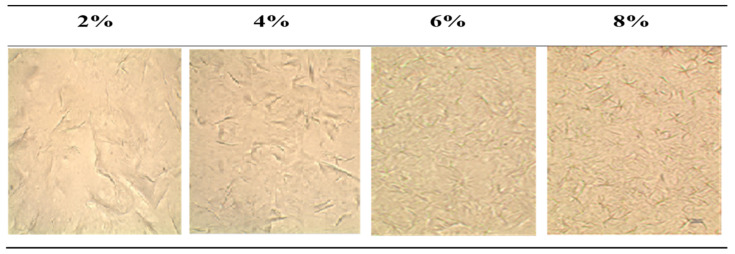
The structure of peanut oil oleogels with yellow beeswax at concentrations: 2, 4, 6 and 8% in the microscopic image at 600× magnification.

The structuring substances in the oleogel form crystal networks that differ in shape and arrangement. By analyzing the crystalline structure of oleogels, it is possible to identify the type of oleogelator used, and also to determine the industrial application of a given oleogel. An adulteration of the oleogel with another structurant can also be detected [20,34]. The average lengths of crystals in this work were 11.03–15.95 nm (Table 1). Natural waxes are a complex mixture of many substances. In beeswax the highest content among other components are esters [43,44]. Hwang et al. [43] reported that the morphology of crystals in “needle” form is a result of high content of esters in the oleogelator. These kinds of crystal forms were also observed in this work. Therefore, it can be assumed that esters, which are present in the chemical composition of yellow beeswax, are responsible for the structure presented in the images of oleogels (Figure 1).

Crystals differed in length, and thin crystals joined together, which was more visible in samples with less wax. At higher concentrations, the crystals were evenly distributed, and in some places the formed aggregates of crystals overlapped each other (Table 1). The increase in the concentration of the structuring substance caused the crystals to be visibly smaller and create a denser network in the image (Figure 1). Similar observations were presented in the work of Pang et al. [26], who also observed a denser network. However, the sizes of the crystals were increasing at higher concentration. This opposite result could be an effect of different preparation and cooling conditions. Rocha et al. [45] reported that oleogels with higher values of hardness were also characterized by a denser oleogelator network. This property was confirmed in the work presented (Table 1, Figure 1).

## 3. Conclusions

Characteristics of oleogels such as microrheological properties, texture, crystal morphology were significantly influenced by the concentration of yellow beeswax. Resistance to centrifugal force was very high at all concentrations of beeswax and no statistically significant differences were obtained. As the content of beeswax increased, the elasticity, macroscopic viscosity, firmness and work of shear of oleogels increased, which was justified by the effect of the more structured network formed when greater oleogelator concentrations are added to the system. Moreover, this work demonstrated that non-invasive optical techniques could be useful in the analysis and objective assessment of physical properties of oleogels.

The obtained results allowed to understand the impact of gelator concentrations in oleogels’ development stages. This work is the first stage of wider research, which has a very significant role in the successful development of a feasible lipid substitute (with health beneficial fatty acids) without compromising product structure and users’ perception, while providing added value to the final product.

## 4. Materials and Methods

### 4.1. Materials

The study material consisted of oleogels that were made of the peanut oil (SCAWAR Sp. z o.o., Warszawa, Poland, country of origin—France) and yellow beeswax, in quantity: 2%, 4%, 6% and 8% (CAN; Norevo GmbH, Hamburg, Germany).

### 4.2. Methods

#### 4.2.1. Oleogel Preparation Method

Oleogels were prepared according to method of Kupiec et al. [18]. The technology of producing oleogels was based on the liquefaction of gelling substances in oil, at 80 °C and mixing during 15 min. A clear solution was obtained and then left to solidify in a thermostatic cabinet (POL-EKO-APARATURA Company Sp. J., Type: ST 2/2 Basic, Wodzisław Śląski, Poland) for 24 h at 20 ± 1 °C. Three samples of each of the four oleogel variants were done.

#### 4.2.2. Oleogel Stability—Centrifuge Method

Oleogel stability was determined by the centrifugal method based on the method of Yılmaz and Öğütcü [46]. Oleogel stability (SO), was calculated based on the formula:

(1) $$\mathrm{SO}=\frac{\left(\mathrm{Mg}\;-\;\mathrm{Mo}\right)}{\left(\mathrm{Mz}\;-\;\mathrm{Mo}\right)\;}\cdot 100\%$$

in which:

Mg—oleogel and test tube weight after centrifugation [g];
Mo—tube weight [g];
Mz—oleogel and test tube weight before centrifugation [g].

#### 4.2.3. Oleogel Microrheological Parameters Evolution Using MS-DWS Method

Microrheology of gels was determined using a Rheolaser Master™ device (Formulaction, L’Union, France), according to the method described by Szymańska et al. [47].

The following rheological parameters of oleogel were determined: macroscopic viscosity index (MVI) (slope nm^−2^), elasticity index (EI) (nm^−2^). The MVI corresponds to the viscosity (in Pa·s) at zero shear, while the EI is directly proportional to the modulus of elasticity G ‘(in Pa) [35,48,49]. The oleogels have been tested 24 h after they were obtained. Measurements were made in triplicate for each sample.

#### 4.2.4. Test for Spreadability of Oleogels

Spreadability tests were performed using a TX.AT plus device (Micro Stable Systems, Godalming, Surrey, UK), at 20 ± 2 °C. The spreadability test consisted of immersing the upper working element—HDP/SR (in the shape of an inverted cone) with a diameter of 4.5 cm in a special container, which was filled with the test sample and ideally suited to the upper working element. The speed at which the upper working element was lowered to get a 1 mm gap between it and container was 3 mm/s. The penetration distance was 23 mm.

The test results were obtained in the form of a force dependence curve [N] and time [s]. Firmness was determined as the highest value on the graph [N], while work of shear was determined as the area below the surface of the force–time graph [N mm], stickiness was determined as the point with the lowest value [N] and work of adhesion was determined as the area over the force–time curve [N mm] [18]. Eight measurements were made for all variants.

#### 4.2.5. Analysis of Oleogel Morphology

Observation of oleogel fat crystals was carried out using a microscope (Delta Optical 100 TP, Mińsk Mazowiecki, Poland) using simple polarization using bright field. A quantity of 50 g of liquefied oleogel was allowed to solidify in a thermostatic cabinet (24 h at 20 ± 2 °C). Next, a small amount of solid sample was placed on a glass microscope slide and was observed at a magnification of 600×. Pictures of samples were taken at ambient temperature (20 ± 2 °C) using a microscope camera (Delta Optical DLT CAM PRO, Minsk Mazowiecki, Poland). The length of fat crystals (average length and minimum and maximum lengths), was determined for 50 measurements for each variant.

#### 4.2.6. Statistical Analysis

Statistical analysis was performed using the Statgraphics Plus 4.1. program (Statgraphics Technologies, Inc., The Plains, VA, USA). Data on the normal distribution were analyzed using a one-way ANOVA variance analysis. Non-parametric data (fatty crystal length) were analyzed using the Kruskal–Wallis rank test. The significance of differences between means was determined based on the Tukey test (*p* ≤ 0.05).

## Figures and Tables

**Table 1:** Oleogels characteristics.

Parameter	Variant *			
	2%	4%	6%	8%
Centrifugal stability [%]	99.02 ^a^ ± 0.93	99.38 ^a^ ± 0.54	99.58 ^a^ ± 0.82	99.56 ^a^ ± 0.32
Firmness [N]	0.854 ^a^ ± 0.02	1.418 ^b^ ± 0.01	7.663 ^c^ ± 0.05	14.010 ^d^ ± 0.02
Stickiness [N]	−1.010 ^a^ ± 0.01	−1680 ^b^ ± 0.01	−9.178 ^c^ ± 0.02	−16.552 ^d^ ± 0.04
Work of shear [N mm]	0.660 ^a^ ± 0.01	2.351 ^b^ ± 0.01	7.469 ^c^ ± 0.02	15.901 ^d^ ± 0.03
Work of adhesion [N mm]	−0.198 ^a^ ± 0.01	−0.354 ^b^ ± 0.02	−1.801 ^c^ ± 0.02	−3.039 ^d^ ± 0.02
Elasticity Index (EI) [nm^−2^]	0.131 ^a^ ± 0.00	0.232 ^b^ ± 0.01	0.594 ^c^ ± 0.00	0.824 ^d^ ± 0.01
Macroscopic Viscosity Index (MVI) [nm^−2^]	0.101 ^a^ ± 0.00	0.311 ^b^ ± 0.00	0.912 ^c^ ± 0.01	1.389 ^d^ ± 0.01
Average length of oleogel crystals [nm]	15.95 ^c^ ± 5.72	12.80 ^a,b^ ± 4.76	10.53 ^a^ ± 4.31	11.03 ^a^ ± 5.02
Minimum (min) length of oleogel crystals [nm]	5.60	6.14	5.89	5.31
Maximum (max) length of oleogel crystals [nm]	40.96	23.04	17.80	20.92

Legend: * 2%—oleogel with 2% yellow beeswax;4%—oleogel with 4% yellow beeswax; 6%—oleogel with 6% yellow beeswax, 8%—organogel with 2% yellow beeswax; ^a,b,c,d^—means in the same column identified by the same letter are not significantly different (*p* ≤ 0.05).

## Data Availability

Not applicable.
